# Assessment of *Canalis Sinuosus* located in maxillary anterior region by using cone beam computed tomography: a retrospective study

**DOI:** 10.1186/s12880-023-01000-x

**Published:** 2023-03-28

**Authors:** Ercin Samunahmetoglu, Mehmet Hakan Kurt

**Affiliations:** Ankara University Faculty of Dentistry, Department of Dentomaxillofacial Radiology, Ankara University, Ankara, Turkey

**Keywords:** Canalis sinuosus, Cone-Beam CT, Three dimensional imaging, Dental implant

## Abstract

**Background:**

The aim of the study is to determine the distribution, location, diameter, and distance measurements of Canalis Sinusosus (CS) in relation with age and sex.

**Methods:**

300 Cone-Beam Computed Tomography (CBCT) images were evaluated. The distance between CS and nasal cavity floor (NCF), buccal cortical bone margin (BCM), alveolar ridge (AR), respectively.The presence of CS smaller than 1 mm, and the diameter of CS larger than 1 mm were determined. Accessory canals (AC) were classified according to their position relative to the teeth.

**Results:**

435 CS with a diameter of at least 1 mm and 142 CS < 1 mm were identified. The most frequently observed location of CS was the region of the right central incisors. The mean diameter of the canals ( CS ≥ 1) was 1.31 ± 0.19 on the right side and 1.29 ± 0.17 on the left side. No gender differences were found in canal diameter were observed (p > 0.05). There was no significant difference between men and women in the distance between CS and the NCF on the right side, and a significant difference was found in the distance of CS-NCF on the left side (p = 0.047). There were no significant differences between age groups in all parameters.

**Conclusion:**

CBCT is a useful tool for identifying CS. Location and diameter of ACs could not be associated with a specific age group or sex.

## Background

The Canalis Sinuosus (CS) is an obscure canal that carries neurovascular bundles called Anterior Superior Alveolar. The name " canalis sinuosus" was introduced by Frederic Wood Jones in 1939 to describe a double-curved bony canal arising from the lateral aspect of the infraorbital canal [1]. As described by Jones [1] and confirmed by other authors, the CS arises from the main stem and is generally located posterior to the middle of the infraorbital canal [2–7]. The first segment of CS runs transversely across the orbital floor before turning medially and merging into a transverse facial segment that runs along the anterior wall of the maxillary sinus [1, 2]. The third, circumferential segment, belonging to CS, runs in a curved course along the piriform opening to reach the anterior maxilla [8]. At the end of its entire course, which is approximately 55 mm long, the CS divides into several narrow accessory canals (ACs) that run toward the teeth and the incisive canal near the nasal septum [8, 9].

Detection of CS using two-dimensional (2D) radiographic methods is very difficult, but can be seen incidentally on periapical radiographs and especially on panoramic radiographs showing a large area. On 2D radiographs, extensions of CS to the anterior maxillary teeth may mimic inflammatory lesions or root resorption when they appear as well-circumscribed circular radiolucencies, especially when superimposed on the roots [10]. On panoramic radiographs, the canal course can be interpreted as a developmental cleft or fracture [11]. Cone beam computed tomography (CBCT) overcomes the limitations of two-dimensional imaging and provides three-dimensional information with submillimeter resolution and prevents superimposition of other structural elements [12]. CBCT can identify the presence of the canal, its location, and its relationship to adjacent structures. CBCT is highly accurate and reproducible in linear measurements and therefore also allows measurement of the canal diameter and the distance of the canal from adjacent structures [13].

Dental implant surgeries are commonly performed treatments to restore esthetics, function, and phonation in the anterior maxilla. Anatomical considerations for successful dental implants in the anterior maxilla include the nasopalatine canal, incisive foramen, and canalis sinuosus, which have neurovascular content [14]. Iatrogenic damage to CS can lead to unpredictable complications such as postoperative pain, paresthesias, and epistaxis that can only be resolved by removal of the implant [14, 15]. There are also case reports from CS, described in the literature, that this may occur in association with an osteolytic lesion of the maxilla or a cyst of the ductus nasopalatinae [16]. As the number of surgical procedures in the anterior maxilla increases, knowledge of CS in this region is important to achieve predictable and safe surgery [7, 17]. Many clinicians have inadequate knowledge of this canal and its potential complications due to some dental procedures. Therefore, this retrospective study was conducted to determine the occurrence, spatial location, diameter, and distance of CS's ACs between the cortical borders of the maxillary alveolus depending on the patient's age and sex.

## Materials and methods

### Study design

This study was designed retrospectively. Ethical approval was obtained from the Local Ethical Board (21/2020). The study protocol complies with the principles outlined in the Declaration of Helsinki. CBCT scans of patients admitted to the Department of Dentomaxillofacial Radiology Department for various diagnostic purposes between January 2013 and February 2021 were retrospectively evaluated. CBCT examinations showing the whole anterior maxillary region with satisfactory diagnostic quality were included. CBCT images were excluded under the following conditions: 1) images with poor diagnostic quality affected by motion or metal artifacts, 2) presence of an impacted tooth, retained rooth, dental implant, or foreign body in the area of interest which prevent the occurence of CS, 3) presence of a syndrome affecting the dentomaxillofacial region (e.g., cleft lip and palate), 4) presence of a metabolic, infectious, or tumour lesion affecting the maxillary region, 5) ongoing orthodontic treatment, 6) surgical procedure or trauma to the anterior maxilla. Based on the inclusion and exclusion criteria, 300 CBCT images were used for the present study.

### Data analysis

**Fig. 1:**
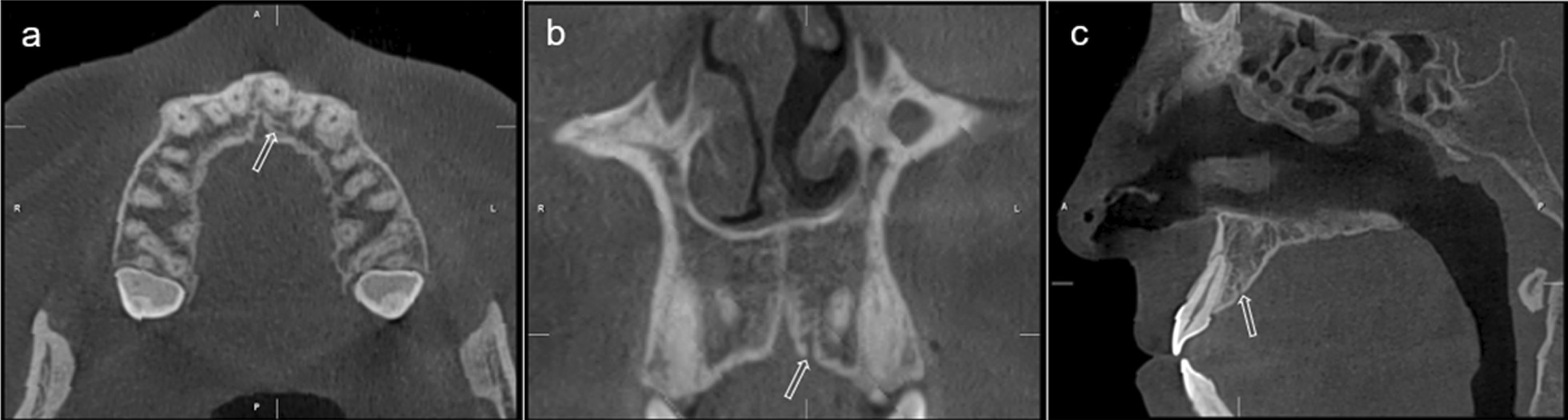
Arrow; accessory branch of the CS adjacent to the palatine of the root of the left central incisor **a** Coronal, **b** Sagittal, **c** Axial images

**Fig. 2:**
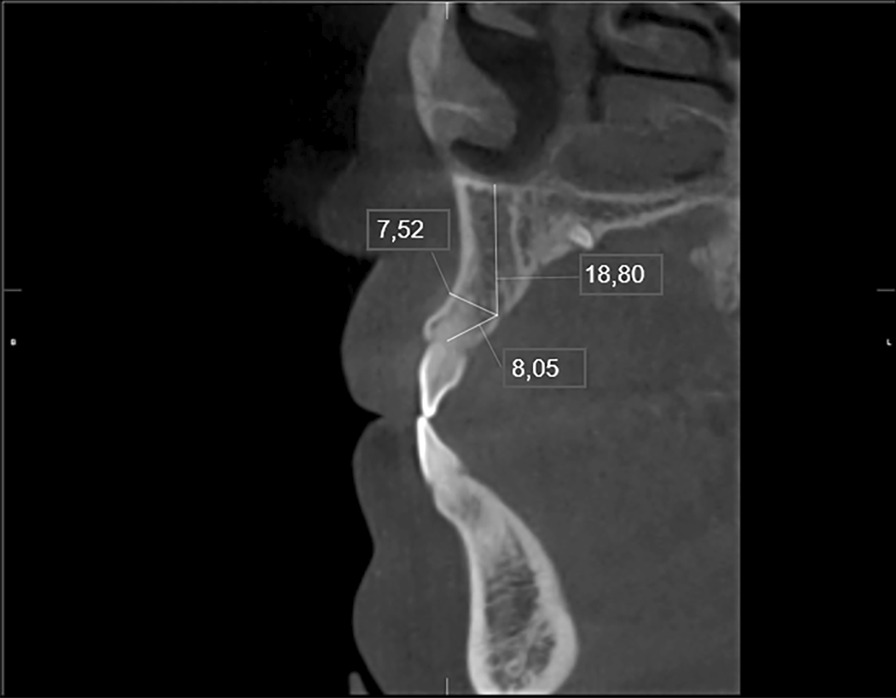
Measurement of the distance from the opening of the accessory canal to the alveolar ridge, to the buccal ridge and to the floor of the nasal cavity in the cross-sectional image

**Fig. 3:**
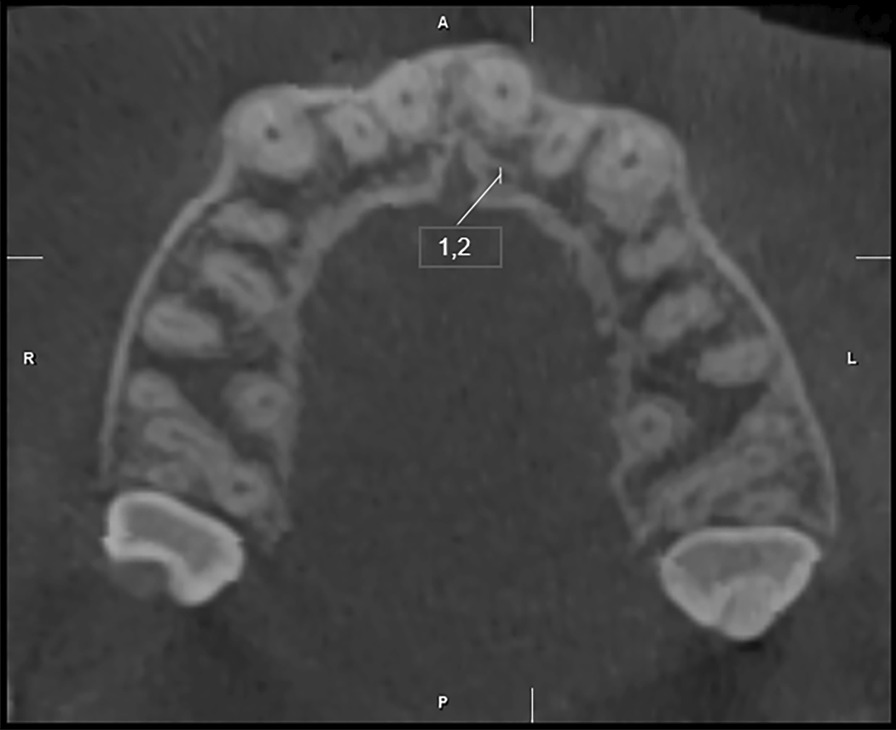
Measurement of the diameter of the accessory canal located in close proximity to the palate of the left central incisor in axial section

The resulting volumetric datasets of each individual were realigned with respect to three orthogonal planes using Planmeca Romexis (3.7; Planmeca, Helsinki, Finland) software. The anatomic axial, coronal, and sagittal planes were aligned with respect to anatomic features or using a reference plane. For the measurements, the head position in the axial view was aligned parallel to the sagittal guide line of the palatal plane (the plane connecting the spina nasalis anterior to the spina nasalis posterior). In the sagittal view, the palatal plane was then aligned parallel to the axial guide line. In the coronal sections, the floor of the nasal cavity was aligned parallel to the horizontal plane. The full volumetric CBCT datasets and the panoramic and cross-sectional reconstructions were analyzed using the tools of Planmeca Romexis (3.7; Planmeca, Helsinki, Finland) software. All measurements and analysis were performed by an oral radiologist. CBCT images were displayed on a 21.3-inch flat panel, color active matrix and thin-film transistor medical monitor with 2048 × 2560 resolution, 11.9 bits, and 75 Hz (NEC MultiSync, Munich, Germany) in a room with dim lighting. Axial, coronal, sagittal, panoramic, and cross-sectional reconstruction images of each patient were analyzed for the presence of CS (Fig. 1). The presence of CS smaller than 1 mm, the diameter of CS larger than 1 mm, the location of CS accessory canals were determined. The ACs were classified according to their position to the teeth: (1) right canine, (2) right lateral incisor-canine, (3) right lateral incisor, (4) right central-lateral incisor, (5) right central incisor, (6) between central incisors (7) left central incisor, (8) left central-lateral incisor, (9) left lateral incisor, (10) lateral incisor-canine, (11) left canine. After locating the canal opening from CS, the distance between the canal opening and (1) nasal cavity floor NCF, (2) the buccal cortical bone margin (BCM), and (3) the alveolar ridge (AR) was measured on cross-sectional images with a slice thickness of 1 mm using the length measurement tool of Planmeca Romexis Viewer software, as shown in Fig. 2. Diameters were determined by measuring the diameter of the canal on the plane where it is closest to the adjacent tooth (Fig. 3).

### Statistical analysis

Data analysis was performed using the SPSS 11.5 program. A p-value of less than 0.05 was accepted as significant. Student's t-test (valid with normal data distribution) or Mann–Whitney U test were used to assess four measurements (the distance between CS and the nasal cavity floor, the distance between CS and the buccal cortical margin, the distance between CS and the alveolar ridge, the diameter of CS) whether there were differences between males and females. Three different distance measurements and the diameter of CS for both sides were analyzed with respect to age groups (under 20 years, 20–29 years, 30–39 years, 40–59 years, and 60 years and older) using the one-way ANOVA test and the Kruskal Wallis H test. Chi-square and Fisher exact tests were performed to determine the presence of CS < 1 mm (CS less than 1 mm in diameter) and its location, whether there was a significant difference between genders and age groups. The paired t-test and Wilcoxon sign test were used to determine the relationship between the distance measurements and the sides. Finally, the Mc-Nemar test was performed to check for the presence of CS < 1 mm if there were differences between the sides.

## Results

This morphometric retrospective study included 181 (60.3%) male and 119 (39.7%) female subjects. The mean age of the 300 participants was 40.02 ± 15.40 (standard deviation) years (range: 10 to 80 years). The distribution of patients by age group was as follows: 26 (8.7%) patients were under 20 years old, 60 (20.0%) were 20–29 years old, 65 (21.7%) were 30–39 years old, 115 (38.3%) were 40–59 years old, 34 (11.3%) were over 60 years old.

A total of 142 CS with a diameter of less than 1 mm and 435 CS with a diameter of at least 1 mm were detected in the anterior maxilla. It was examined that the presence of CS < 1 mm for the both right and left sides, and a significant difference was found between the two sides (p = 0.020). The mean diameter of the canals ( CS ≥ 1) was 1.31 ± 0.19 on the right side and 1.29 ± 0.17 on the left side. There was no significant difference between the measurements of the right and left sides in terms of the diameter of CS ≥ 1 mm (p = 0.212). The most frequently observed location of CS was the right central incisors, followed by the left central-lateral incisor region. The least frequent location was the left canine region (Table 1).

**Table 1:** Distribution of CS with different diameters in the maxilla anterior and the comparision of the four measurements and location between right and left sides (NCF:distance between CS and nasal cavity floor, BCM:distance between CS and buccal cortical bone margin, ACr:distance between CS and alveolar crest, CS ≥ 1 mm: diameter of CS measured at least 1 mm)

Variables	Right side	Left side	p value
Presence of CS < 1 mm, n(%)			
Absent	216 (72.2)	239 (80.2)	0.020^a^
Present	83 (27.8)	59 (19.8)	
Diameter of CS ≥ 1 mm			
Mean ± SS	1.31 ± 0.19	1.29 ± 0.17	0.212^a^
Median (Min.-Max.)	1.26 (1.00–1.79)	1.26 (1.00–1.79)	
Location, n(%)			
Intercentral	29 (9.9)	22 (7.6)	0.622^b^
Central Incisors	93(31.8)	71 (24.3)	
Central-Lateral Incisors	60 (20.6)	83 (28.5)	
Lateral Incisors	60 (20.6)	73 (25.1)	
Lateral Incisors-Canine	29 (9.9)	29 (10.0)	
Canine	21 (7.2)	13 (4.5)	
ACs-NCF			
Mean ± SS	11.87 ± 3.61	11.81 ± 4.13	0.632^b^
Median (Min.-Max.)	12.01 (3.42–21.00)	11.91 (2.04–21.15)	
ACs-BCM			
Mean ± SS	4.60 ± 1.69	4.73 ± 1.75	0.805^b^
Median (Min.-Max.)	4.81 (0.89–8.41)	4.83 (0.80–12.01)	
ACs-ACr			
Mean ± SS	7.77 ± 3.24	7.93 ± 3.79	0.443^b^
Median (Min.-Max.)	7.22 (1.26–8.09)	7.38 (0.89–21.85)	

^a^ Mc-Nemar and Paired-t test, ^b^ Wilcoxon sign test and Fisher exact test

The distance measurements of the canal openings and the diameter of the ACs were shown in Table 2. There was no significant difference between males and females in the distance of ACs-BCM, ACs-AR measurements on both sides (p > 0.05). As with the distance measurements, no significant difference was found between males and females for the canal diameters on both sides (p > 0.05). While there was no significant difference between males and females in the distance of ACs-NCF measurement on the right side (p = 0.081), there was a significant difference in the distnace of Acs-NCF on the left side (p = 0.047).

**Table 2:** Comparison of the distance measurements and diameter of ACs on the right and left side by gender

Sides	Measurements	Male		Female		
		Mean ± SD	Median (Min.-Max.)	Mean ± SD	Median (Min.-Max.)	p value
Right	ACs-NCF	12.17 ± 3.63	12.23 (3.42–21.00)	11.42 ± 3.56	11.27 (4.47–19.22)	0.081^a^
	ACs-AR	7.82 ± 3.29	7.35 (1.35–18.09)	7.70 ± 3.16	7.07 (1.26–18.09)	0.899^b^
	ACs-BCM	4.54 ± 1.64	4.76 (0.89–8.41)	4.69 ± 1.77	4.82 (0.89–8.41)	0.367^b^
	CS ≥ 1 mm	1.32 ± 0.20	1.26 (1.00–1.79)	1.30 ± 0.18	1.26 (1.00–1.79)	0.591^b^
Left	ACs-NCF	12.20 ± 4.02	12.04 (2.04–21.15)	11.21 ± 4.25	11.39 (2.04–20.83)	0.047^a^
	ACs-AR	8.01 ± 3.98	7.34 (0.89–21.85)	7.81 ± 3.47	7.38 (0.89–17.20)	0.993^b^
	ACs-BCM	4.85 ± 1.75	4.88 (0.80–12.01)	4.56 ± 1.74	4.59 (0.80–12.01)	0.163^a^
	CS ≥ 1 mm	1.30 ± 0.17	1.26 (1.02–1.79)	1.28 ± 0.16	1.26 (1.00–1.70)	0.926^b^

^a^ Student-t test, ^b^ Mann-Whitney U test

As shown in Table 3, ACs were mostly detected in the central incisor region on the right side in both genders, while it was most detected in the central lateral incisor region on the left side. There was no significant relationship between the presence of CS < 1 mm and gender on both the right and left sides (p = 0.785, p = 0.685, respectively).

**Table 3:** Comparison of canals location and presence of CS < 1 mm with gender

Side		Male		Female		
		n	%	n	%	p value
Right	Presence of CS < 1 mm					
	Absent	129	71.7	87	73.1	0.785^a^
	Present	51	28.3	32	26.9	
	Location					
	Canine	13	7.4	8	6.8	0.398^a^
	Lateral Incisors- Canine	18	10.3	11	9.4	
	Lateral Incisors	31	17.7	29	24.8	
	Central-Lateral Incisors	42	24.0	18	15.4	
	Central Incisors	56	32.0	37	31.6	
	Intercentral	15	8.6	14	12.0	
Left	Presence of CS < 1 mm					
	Absent	143	79.4	96	81.4	0.685^a^
	Present	37	20.6	22	18.6	
	Location					
	Intercentral	14	7.9	8	7.0	0.756^a^
	Central Incisors	44	24.8	27	23.7	
	Central-Lateral Incisors	47	26.6	36	31.6	
	Lateral Incisors	44	24.8	29	25.4	
	Lateral Incisors-Canine	21	11.9	8	7.0	
	Canine	7	4.0	6	5.3	

^a^ Chi-square test

It was examined whether there was a statistically significant difference between the age groups and the distance measurements. There was no significant difference between the age groups in the distance of ACs-NCF, ACs-AR, ACs-BCM on the right and left sides. No correlation was found between the age groups and the canal diameter (Table 4). In addition, no significant correlation was found between the presence of CS less than 1 mm in diameter and the canal locations among age groups on both sides (Table 5).

**Table 4:** Comparison of the distance measurements and diameter of ACs on the right and left side according to age groups

	Age groups	Right			Left		
		n	Mean ± SD	p value	n	Mean ± SD	p value
ACs-NCF	< 20	26	10.55 ± 4.02	0.273^a^	24	11.83 ± 4.07	0.983^a^
	20–29	59	11.68 ± 3.83		57	11.72 ± 4.16	
	30–39	61	11.77 ± 3.68		65	11.96 ± 3.77	
	40–59	114	12.22 ± 3.33		111	11.68 ± 4.32	
	≥ 60	32	12.25 ± 3.64		34	12.11 ± 4.37	
ACs-AR	< 20	26	7.51 ± 2.79	0.311^b^	24	7.85 ± 3.37	0.293^b^
	20–29	59	8.30 ± 3.30		57	8.32 ± 3.92	
	30–39	61	8.09 ± 3.14		65	8.57 ± 3.95	
	40–59	114	7.55 ± 3.21		111	7.64 ± 3.69	
	≥ 60	32	7.20 ± 3.71		34	7.07 ± 3.79	
ACs-BCM	< 20	26	4.71 ± 2.32	0.320^a^	24	5.23 ± 1.79	0.268^b^
	20–29	59	4.46 ± 1.70		57	5.09 ± 2.03	
	30–39	61	4.40 ± 1.62		65	4.51 ± 1.60	
	40–59	114	4.84 ± 1.60		111	4.61 ± 1.60	
	≥ 60	32	4.30 ± 1.47		34	4.65 ± 1.88	
CS ≥ 1 mm	< 20	22	1.24 ± 0.15	0.357^b^	21	1.27 ± 0.17	0.415^b^
	20–29	48	1.30 ± 0.17		51	1.31 ± 0.17	
	30–39	53	1.33 ± 0.21		59	1.31 ± 0.17	
	40–59	104	1.32 ± 0.19		99	1.27 ± 0.16	
	≥ 60	26	1.34 ± 0.21		32	1.31 ± 0.16	

^a^ one way ANOVA test, ^b^ Kruskal Wallis H test

**Table 5:** Evaluation of the presence of CS with a diameter of less than 1 mm and canal locations according to age groups

		Age groups					p value
		< 20	20–29	30–39	40–59	≥ 60	
		n (%)	n (%)	n (%)	n (%)	n (%)	
Presence of CS < 1 mm (Right)	Absent	21 (80.8)	43 (71.7)	41 (64.1)	87 (75.7)	24 (70.6)	0.434^a^
	Present	5 (19.2)	17 (28.3)	23 (35.9)	28 (24.3)	10 (29.4)	
Location (Right)	Intercentral	1 (3.8)	4 (6.8)	5 (8.2)	7 (6.1)	4 (12.5)	0.803^b^
	Central Incisors	2 (7.7)	8 (13.6)	6 (9.8)	11 (9.6)	2 (6.3)	
	Central-Lateral Incisors	6 (23.1)	13 (22.0)	9 (14.7)	21 (18.4)	11 (34.4)	
	Lateral Incisors	5 (19.2)	11 (18.6)	14 (23.0)	24 (21.1)	6 (18.7)	
	Lateral Incisors-Canine	11 (42.4)	17 (28.8)	22 (36.1)	35 (30.8)	8 (25.0)	
	Canine	1 (3.8)	6 (10.2)	5 (8.2)	16 (14.0)	1 (3.1)	
Presence of CS < 1 mm (Left)	Absent	21 (84.0)	49 (81.7)	52 (80.0)	87 (76.3)	30 (88.2)	0.596^a^
	Present	4 (16.0)	11 (18.3)	13 (20.0)	27 (23.7)	4 (11.8)	
Location (Left)	Intercentral	0 (0.0)	7 (12.3)	4 (6.2)	10 (9.0)	1 (2.9)	0.605^b^
	Central Incisors	7 (29.2)	17 (29.8)	11 (16.9)	28 (25.2)	8 (23.5)	
	Central-Lateral Incisors	10 (41.6)	12 (21.1)	21 (32.3)	30 (27.0)	10 (29.4)	
	Lateral Incisors	6 (25.0)	10 (17.5)	18 (27.7)	29 (26.2)	10 (29.4)	
	Lateral Incisors-Canine	1 (4.2)	6 (10.5)	8 (12.3)	11 (9.9)	3 (8.9)	
	Canine	0 (0.0)	5 (8.8)	3 (4.6)	3 (2.7)	2 (5.9)	

^a^ Chi-square test, ^b^ Fisher-exact test

## Discussion

There were many descriptions of specific parts of CS in textbooks/literature ages ago and the first to describe the terminal part of the CS was Macalister [1, 18]. Nowadays, the CS and the terminal branches extending the alveoli of the maxilla are also described in modern anatomy textbooks [8, 19]. However, some practitioners have insufficient knowledge about this canal and there are still misunderstandings about whether the canal is an anatomical structure or a variation [20, 21]. The distribution and diameter of the canal extensions of the CS in the anterior maxilla vary from person to person and are not type-specific [6]. These additional and inferior branches of CS are called accessory canals [22].

To avoid surgical errors, anthropometric measurements and morphometric analysis of anatomic variations are essential to achieve the intended treatment plan. Incomplete or neglected preoperative examinations may lead to complications. Therefore, knowledge of the potential risks and factors that may lead to complications is critical. The studies and limited information, which are typical of cross-sectional research and less common in longitudinal studies, exhibit heterogeneity in the literature. Clinical, methodological, and statistical factors may have contributed to the variability. The literature clearly demonstrated methodological diversity, in particular [23]. As a result, there is no standardized approach or information, and more CS data is required.

The limited number of studies on CS have reported quite variable rates for the prevalence of this structure. The prevalence of CS varies from 15.7% to 100% in studies using different investigational methods in different populations with different sample sizes [4, 5]. The prevalence determined when canals with a diameter of at least 1 mm are considered is almost half of the prevalence determined regardless of canal diameter [17]. It has been reported that the visibility of accessory canals of CS less than 1 mm in diameter is reduced. Detection of CS less than 1 mm in diameter with CBCT depends on the voxel size [9], which is one of the many factors (nominal pixel size of the detector, beam projection geometry, scatter radiation, motion blur of the detector, fill factor, focal spot size, number of base images, and reconstruction algorithm) that affect spatial resolution [24]. The smallest voxel size that the detector may vary from one manufacturer to another [25]. The limitation of the devices to visualize CS with a diameter of < 1 mm and the inexperience of the clinician to detect these canals lead CS to misinterpretation of CS as a rare anatomical variation.

Temmerman et al. [26] reported the prevalence, diameter and length of a recently detected canal palatal to the upper canine and this canal in the upper canine was observed in 32.9% of cases. In the study by Oliveira Santos et al. [5], it was found that the accessory canals of CS were observed in regions other than the canine region. The distribution of accessory canal openings in the anterior maxilla can be classified according to their location in relation to the teeth, incisive foramen, and spatial position (palatal, transverse, and buccal) [9, 27, 28]. Termination of CS was more common in the anterior region of the maxilla, and more specifically, it was more frequently observed palatally in the incisor and canine regions [3]. The vast majority of neurovascular disorders caused by iatrogenic injury to the CS occur in the dentoalveolar region of the anterior maxilla during dental implant surgery. Therefore, the location of CS relative to the teeth was identified in our study and was most frequently found in the region of the right central incisor. A comprehensive examination should be done into the clinical significance of the accessory canal's location. When frequency and opening location are combined, the area surrounding the maxillary incisors appears to provide higher risks from accessory canals for implant insertion. To prevent neurovascular damage, clinicians must be careful when placing implants in the alveolar bone, especially when it comes to their orientation and depth.

In the present study, there was no significant correlation between the presence of ACs < 1 mm and gender. In the study by Orhan et al. [27], there was a tendency for a higher incidence of ACs in females than in males, which was also observed in the study by Oliveira Santos et al. [5]. However, no statistically significant gender difference was found. In the study by Gurler et al. [4], a higher frequency of CS was observed in females. They also reported that these results were not accurate because the number of females in the study was almost twice that of males and they also detected CS in only a few samples. In the study by Sekerci et al. [29], ACs were observed more in females (29.5%) than in males (15.7%). Ghandourah et al. [30], Tomrukçu et al. [31] reported that there was no significant difference between the presence of AC and gender, which is consistent with published results and our findings, except Machado et al. [9].

Regarding the presence of CS and age, Oliveira-Santos et al. [5], von Arx et al. [24], Wanzeler et al. [6], Aoki et al. [21], and Anatoly et al. [28] reported that no statistically significant difference was found between the prevalence of CS and age groups. In the study by Sekerci et al. [29], a steady increase in occurrence in older age groups in the pediatric population was demonstrated. Machado et al. [9] found that there was a weak correlation between the number of CS and age. Orhan et al. [27] in the Turkish population and Ghandourah et al. [30] in the German population reported a higher incidence in older age groups compared to younger adults. In the study by Baena-Caldas et al. [20], conducted in a population with a wide age range (9 to 93 years), a steady increase in incidence was demonstrated in older patients, although there was a decrease after the age of 71 years. Tomrukcu et al. [31] reported that no statistically significant difference was found between age groups in terms of canal diameter, except for the 80–89 years old group. They also reported that the number of patients aged 80–89 years was only two and CS was detected in two patients (100%). In our study, no significant correlation was found between the presence of a CS < 1 mm on the right side and age groups. In addition, there was no significant correlation between the presence of CS < 1 mm on the left side and age groups.

In the study by Temmerman et al. [26], the diameter of the canals in the upper canine region was measured on the axial slices where it exited the maxilla, to the nearest 0.1 mm. These canals were reported to have an average diameter of 1.23 mm (range: 0.5–7.7 mm). Oliveira-Santos et al. [5] reported that the average diameter of the CS openings in the anterior palate was 1.4 mm (range: 1.1 mm-1.9 mm). Von Arx et al. [24] reported that the average diameter of CSs larger than 1 mm was 1.31 mm (median 1.23 mm, range 1.01–2.13 mm, standard deviation ± 0.26 mm). Şekerci et al. [29] reported the mean canal diameter as 1.12 mm (range 1–1.7 mm, standard deviation ± 0.26 mm) and the diameters were determined by measuring the palatal opening of the canal with a diameter of at least 1 mm. Machado et al. [9] determined the diameter of each canal (at least 1 mm in diameter) at the median distance of its total length. They detected 195 CS with a diameter of at least 1.0 mm (20.0% of all CS); the mean diameter of these CSs was reported to be 1.19 mm (median 1.15 mm, range 1.00–2.58 mm, standard deviation ± 0.22 mm). In the study by Gurler et al. [4], the mean diameter of CS was 1.37 mm (range: 0.75–2.25 mm), and canals with a diameter of less than 1 mm were also included in this study. In the study by Tomrukcu et al. [31], the mean diameter of ACs considered with a minimum of 0.5 mm was 1.30 mm (range 0.57–2.88 mm, SD ± 0.44 mm). The diameters were determined by measuring the palatal opening of the ACs. Although the association between canal diameter and complication prevalence is unclear, it is possible to emphasize the fact that the diameter of the neurovascular bundle, which may increase the risk of surgical complications, specifically the amount of bleeding. Beside, increasing the diameter of canal may lead misdiagnosis for periapical lesions on conventional radiography. Thus, in the present study, the largest diameter of the CS was measured at the point where it was closest to the teeth on axial slices. The canals with diameter less than 1 mm were not considered. The mean diameter of ACs in the present study was 1.26 mm (range: 1 mm-1.79 mm). From the literature, the mean diameters of the foramina of the ACs ranged from 1.12 mm to 1.6 mm. This difference may be related to the quality or thickness of the image slices, the percentage of canals with a diameter of at least 0.5 mm (if included), consideration of the diameter of the palatal opening of the ACs, and not the diameter of the canal. No statistically significant difference was found between the diameter of the ACs and gender. This result is in contrast to the results of Von Arx et al. [24], Machado et al. [9], Gurler et al. [4], Tomrukcu et al. [31], who found that the canal diameter was larger in males than in females.

During endodontic surgery, particularly during the curettage of the periapical lesion, the neurovascular systems within the CS may be affected. Although there is not enough clinical evidence to say if increased bleeding during endodontic surgery is caused by CS injury, insufficient vasoconstrictors, or a combination of the two, it is nonetheless therapeutically important given the short distance. The potential for nerve tissue injury to result in pain and/or paresthesia noted in the literature [17, 32, 33]; as a result, some of the unidentified face discomfort experienced by a patient who has had surgery may be caused by damage to the CS.

In surgical procedures, the anterior maxilla, generally regarded as a relatively safe location for implant insertion, recommends additional consideration. In order to accurately detect and locate accessory canals and reduce the risk of neurovascular damage, preoperative CBCT testing should be consistently carried out. When performing maxillary surgery, the distance between the canal opening and the alveolar crest may be used as a personal indicator to pinpoint the ideal location for the implantation of dental implants and the harvesting of bone [17]. Wanzeler et al. [6] reported that the mean distance between the terminal part of CS and the alveolar ridge area was 25.82 ± 6.7 mm (maximum 24.8 mm, minimum 0 mm) in males and 14.97 ± 5.37 mm (maximum 12.98 mm, minimum 0 mm) in females. Gurler et al. [4] reported that the mean distance between the terminal part of the CS and the alveolar ridge was 16.81 mm (range 0–23.5 mm). In the present study, the mean distance between the CS and the alveolar crest in females was 7.70 mm on the right side and 7.81 mm on the left side. Similarly, the mean distance between CS and the alveolar crest in males was 7.82 mm on the right side and 8.01 mm on the left side. In the present study, the distance between the palatal opening of the CS and the alveolar crest was measured rather than the distance between the terminal part of the CS and the alveolar crest, which may explain the difference in mean distance. In the study by Wanzeler et al. [6], the distance between the end of CS and the alveolar crest was greater in males than in females, and there was a statistically significant difference related to gender. This finding is in contrast to the results of Tomrukcu et al. [31], who found a greater distance between the terminal part of CS and the region of the alveolar ridge in females than males. In the present study, there was no statistically significant difference between men and women on both the right and left sides for all distance measurements of CS. The reason for the lack of agreement is that there are many factors that affect the dimension of the alveolar bone including; individual anatomical differences, racial differences, age, and early tooth loss. In addition, the alveolar process undergoes structural and compositional changes over time [7].

### Limitation of the study

In the present study, the group of child to early adulthood is not categorized to smaller groups i.e. child, adolescent, teenager, adult. Moreover, the distrubution between age groups are not equal.

## Conclusion

The presence, location, and diameter of the ear canals of CS cannot be associated with a specific age group or gender. In addition, there is a large age range in the child to early adulthood group (< 20 years). It should be categorized to smaller groups in future studies. For pre-surgical procedures performed in the anterior maxilla, CS should be examined with CBCT, which provides three-dimensional information and applicability of the results in clinical practice. The evidence and morphological information supplied may enhance success rates while decreasing the risks of accidents and problems. More anatomic research is needed to learn more about the CS.

## Data Availability

The data that support the fndings of this study are available on request from the corresponding author. The data are not publicly available due to information that could compromise the privacy of research participants.

## Abbreviations

CS: Canalis sinuosus
CBCT: Cone Beam Computed Tomography
2-D: Two-dimensional
ACs: Accessory canals
NCF: Nasal cavity floor
AR: Alveolar ridge
BCM: Buccal cortical bone margin
